# Monolithic integration of a lithium niobate microresonator with a free-standing waveguide using femtosecond laser assisted ion beam writing

**DOI:** 10.1038/srep45610

**Published:** 2017-03-30

**Authors:** Zhiwei Fang, Yingxin Xu, Min Wang, Lingling Qiao, Jintian Lin, Wei Fang, Ya Cheng

**Affiliations:** 1 State Key Laboratory of High Field Laser Physics, Shanghai Institute of Optics and Fine Mechanics, Chinese Academy of Sciences, Shanghai 201800, China; 2 University of Chinese Academy of Sciences, Beijing 100049, China; 3 School of Physical Science and Technology, ShanghaiTech University, Shanghai 201210, China; 4 State Key Laboratory of Modern Optical Instrumentation, College of Optical Science and Engineering, Zhejiang University, Hangzhou 310027, China; 5 State Key Laboratory of Precision Spectroscopy, East China Normal University, Shanghai 200062, China; 6 Collaborative Innovation Center of Extreme Optics, Shanxi University, Taiyuan, Shanxi 03006, China

## Abstract

We demonstrated integrating a high quality factor lithium niobate microdisk resonator with a free-standing membrane waveguide. Our technique is based on femtosecond laser direct writing which produces the pre-structure, followed by focused ion beam milling which reduces the surface roughness of sidewall of the fabricated structure to nanometer scale. Efficient light coupling between the integrated waveguide and microdisk was achieved, and the quality factor of the microresonator was measured as high as 1.67 × 10^5^.

Lithium niobate (LN) microresonators have attracted much attention for their broad range of applications in optical signal processing, quantum electrodynamics and optomechanics^1^,^2^,^3^,^4^,^5^,^6^,^7^,^8^,^9^. This is mainly due to the excellent material properties of lithium niobate such as a broad transmission window, large nonlinear optical coefficients, and a large electro-optic tunability. Particularly, the recent advance in the fabrication of high-Q lithium niobate microresonators has promoted the quality factor of such microresonators to 10^5^~10^6^ 3,4,5,6,7,8,9. This is enabled by patterning a lithium niobate thin film bonded onto 2 μm-thickness SiO_2_ with either focused ion beam or reactive ion dry etching^3^,^5^. An additional chemical wet etching in hydrofluoride (HF) acid will selectively remove the SiO_2_, resulting in a free-standing microdisk that can serve as a whispering-gallery mode (WGM) microresonator. To utilize the high-Q microresonators, light has to be coupled into and out from the microresonator. This is typically achieved using external fiber taper critically coupled to the microdisk. For many applications, it would be desirable to replace the external fiber tapers with monolithically integrated waveguides, thereby the devices can be miniaturized and will be more stable against the disturbance from the environment.

Here, we show that the above-mentioned monolithic integration can be realized with femtosecond laser assisted ion beam writing (FLAIBW), a technique we developed in 2014 for producing high-Q lithium niobate microresonators^3^,^4^. The advantages of this technology include the followings. First, both femtosecond laser direct writing and focused ion beam (FIB) writing are maskless fabrication techniques, which provide the flexibility to achieve rapid prototyping of various kinds of devices. Second, the femtosecond laser is much more efficient than the FIB in terms of the removal rate of material, whilst it is of relatively low fabrication resolution on the order of 1 μm as compared to that of FIB. The combination of two approaches allows a high fabrication efficiency mainly determined by the femtosecond laser direct writing and a high fabrication resolution completely determined by the FIB writing. At last, the femtosecond laser has been widely used in fabricating photonic structures in various crystals thanks to the unique 3D fabrication capability in transparent materials^10^,^11^,^12^,^13^,^14^. The high-precision 3D microfabrication is realized by irradiating inside transparent materials with tightly focused femtosecond laser pulses of which the ultrashort pulse duration and ultrahigh peak intensity together can lead to efficient confinement of the nonlinear interactions within the focal volume. Therefore the femtosecond laser direct writing can easily incorporate other functionalities such as microfluidics and microelectrodes into the devices^15^,^16^,^17^.

## Results and Discussion

**Figure 1:**
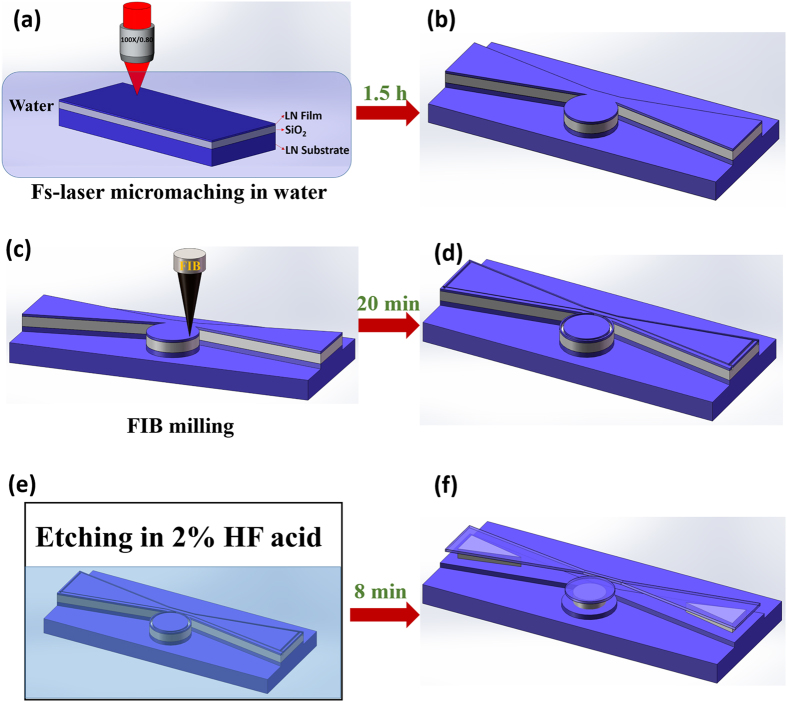
The processing flow of fabricating an on-chip LN microresonator integrated with a waveguide is illustrated. (**a**,**b**) Formation of LN microresonator with the integrated waveguide using femtosecond laser microfabrication. (**c,d**) Focused ion beam (FIB) milling to smooth the periphery of the structure fabricated by femtosecond laser direct writing as shown in (**b**). (**e,f**) Chemical wet etching of the sample undergone the FIB milling to form the freestanding LN microdisk resonator and waveguide.

The technical details on the fabrication of on-chip LN microresonator integrated with a waveguide using FLAIBW can be found in Methods. Briefly speaking, a commercially available ion-sliced Z-cut LiNbO_3_ (LN) thin film with a thickness of 900 nm (NANOLN, Jinan Jingzheng Electronics Co., Ltd) was chosen for fabricating the on-chip LiNbO_3_ microdisk resonators. The LN thin film was bonded on a 0.5-mm thick LN substrate sandwiched by a SiO_2_ layer with a thickness of 2 μm^18^,^19^,^20^. The procedures of fabricating the integrated device are schematically illustrated in Fig. 1. First, a microresonator-waveguide system was fabricated by ablating the LN substrate immersed in water using tightly focused femtosecond laser pulses, as shown in Fig. 1(a). The height of the patterned microstructure is 10 μm, as schematically illustrated in Fig. 1(b). The femtosecond laser fabrication took about 1.5 hrs. Next, the periphery of the LN microdisk and waveguide were smoothed using focused ion beam (FIB) milling, as illustrated in Fig. 1(c). In this step, the microdisk was separated from the waveguide by FIB, as shown in Fig. 1(d). The FIB milling was completed in 20 min. Finally, chemical wet etching, which selectively removes the SiO_2_ underneath the LN thin film to form free-standing LN microdisk resonator and membrane waveguide, was performed by immersing the fabricated structure in a solution of 2% hydrofluoric (HF) for 8 minutes, as shown in Fig. 1(e). The SiO_2_ layer was partially preserved to support the LN microdisk and waveguide. The diameter of the LN microdisk is 20 μm. It took about 2 hrs in total to produce the integrated device. More details of the process flow of fabricating the LN microresonator can be found in [3] and [4].

**Figure 2:**
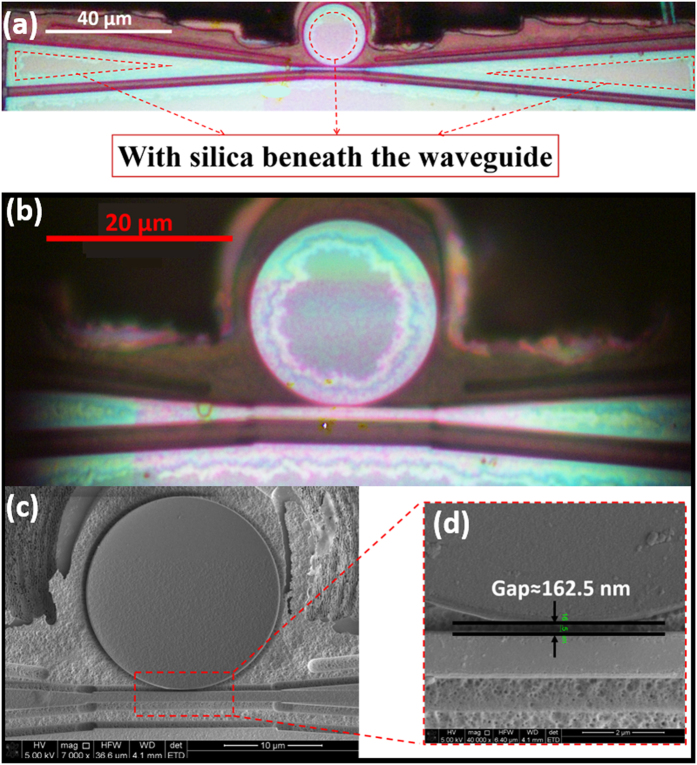
(**a**) Top view optical micrograph of the entire integrated device. (**b**) Close-up view optical micrograph of the microdisk coupled with the waveguide. (**c**) SEM image of the LN microdisk coupled to the LN waveguide and (**d**) closed-up view of the coupling area with the gap between the LN microdisk and waveguide of 162.5 nm width.

Figure 2(a) shows the overview optical micrograph of the fabricated microresonator-waveguide system. It can be seen that a thin waveguide has been fabricated near the microresonator. The waveguide is tapered at its two ends for efficient light coupling into and out of waveguide. For the middle part of the waveguide which is coupled with the microresonator, the width is approximately 1 μm. For achieving a higher coupling efficiency, waveguide with a width smaller than 1 micron may provide better performance. However, from the fabrication point of view, waveguide of a width of 1 micron is relatively easy to fabrication and the fabricated structure is more mechanically stable. Meanwhile, an efficient coupling can still be maintained with this parameter. The areas enclosed in the dashed-line triangles are supported by the SiO_2_ which survived from the selective chemical etching, thus the waveguide can be fixed on the substrate. A close-up view micrograph shown in Fig. 2(b) provides more details of the fabricated structure, showing the smooth edges of the waveguide and microresonator as a result of FIB milling. Furthermore, the scanning electron micrograph (SEM) in Fig. 2(c) shows that there is a small gap between the microdisk resonator and waveguide. The width of the gap is measured to be 162.5 nm, as indicated in Fig. 2(d). Such a narrow gap is important for achieving the near critical coupling condition which is necessary for obtaining the high-Q factors.

**Figure 3:**
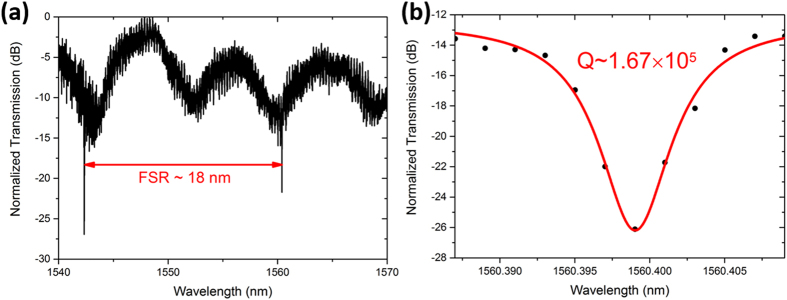
(**a**) Normalized transmission spectrum of the integrated device. (**b**) The Lorentzian fitting shows a Q-factor of 1.67 × 10^5^.

Figure 3(a) shows the measured transmission spectrum of the integrated microresonator-waveguide system (for details, see Methods). Based on a Lorentzian fitting in Fig. 3(b), the Q factor was determined to be 1.67 × 10^5^. It is noteworthy that the Q factor of the integrated LN microdisk-waveguide system is approximately two orders of magnitude higher than the Q-factor previously demonstrated with the integrated LN microring-waveguide system^1^,^21^. As the LN film has very smooth top and bottom surfaces, the major factor that spoils the Q-factor of resonator is the scattering from the sidewall of microdisk whose roughness is determined by the quality of FIB milling. In our case, finely-tuned FIB milling process ensures that the side wall has a roughness <5 nm, so that scattering is strongly suppressed. However, the previous micro-ring devices were fabricated by dry etching, thus the sidewall roughness might be higher. Besides, for the micro-ring structure, both the outer and inner sidewalls can introduce optical loss, which further brings down the Q-factor.

**Figure 4:**
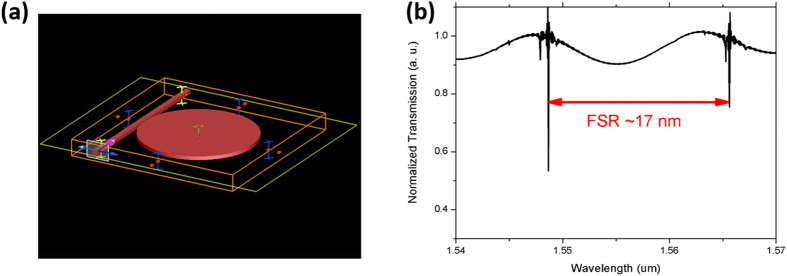
(**a**) Illustration of the model structure used in the simulation. (**b**) Normalized transmission spectrum of the integrated device for an input TE beam.

We further performed the theoretical analysis using a Finite Difference Eigenmode solver (MODE Solutions, Lumerical), which numerically solves Maxwell equation directly in time domain and offers both high precision and acceptable computing time. Figure 4(a) presents the model structure used in the simulation, of which all the parameters are the same as that in the experiment. In the model structure, the lithium niobate micordisk has a thickness of 900 nm and a diameter of 20 μm. The refractive index of lithium niobate is chosen with a Z cut birefringence, which is also consistent with the experimental situation^22^,^23^. The waveguide coupled to the microdisk has a width of 1 μm and a length of 20 μm. In our simulations, both TE and TM beams with fundamental spatial modes propagated from one end of the waveguide, and the transmission spectra were monitored from the other end of the waveguide after the beams in the waveguide had interacted with the high-Q microresonator. By tuning the width of the gap between the waveguide with micodisk, we found that the critical coupling is achieved for a width of 125 nm for TE mode and a width of 100 nm for TM mode. It was found that the calculated transmission spectrum for the TE beam, as shown in Fig. 4(b), can faithfully reproduce the measured result in Fig. 3(a). The free spectrum range (FSR) determined by the spectrum in Fig. 4(b) is 17 nm, which is very close to the measured FSR of 18 nm as indicated in Fig. 3(a). The small difference could be attributed to the fact that it is difficult to precisely determine all the parameters of the microdisk and the waveguide, such as the thickness and the transverse dimensions from an experimental point of view.

## Conclusion

To conclude, we have demonstrated an approach for monolithically integrating an optical microresonator with an optical waveguide on lithium niobate substrate. Our technique, which is termed as FLAIBW, combines femtosecond laser direct writing and FIB writing. Therefore, FLAIBW offers advantages including high fabrication efficiency (in comparison with that of FIB writing) and ultra-high fabrication resolution. The fabricated device shows a quality factor above 10^5^. It should be mentioned that the top surface of the LN film is well polished by manufacturer, and the roughness is claimed to be sub-nanometer. The sidewalls of the LN disk and waveguide are very smooth after FIB milling, and the roughness is estimated as <5 nm via SEM investigation. We think that by reducing the roughness induced by FIB milling, the Q-factor of the microresonator can even be further improved. The technique can also be extended for coupling multiple remotely distributed microresonators using the bus waveguide. We expect that a lot of nonlinear optical effects could be realized in the integrated LN microresonator, including second harmonic generation, frequency comb generation, four wave-mixing, and so on. The major challenge to realize efficient nonlinear optical processes is to achieve phase matching for the pump and signal waves. The integrated devices are attractive particularly from an application point of view because of the enhanced compactness and stability.

## Methods

### Fabrication of on-chip LN microresonator-waveguide system

A Ti: sapphire femtosecond laser source (Coherent, Inc., center wavelength: 800 nm, pulse width: 40 fs, repetition rate: 1 kHz) was used for fabricating the on-chip LN microresonator integrated with a waveguide. A variable neutral density filter was used to tune the average power of the laser beam. In the femtosecond laser direct writing, an objective lens (100×/NA 0.80) was used to focus the beam down to a ~1 μm-diameter focal spot. The sample could be arbitrarily translated in 3D space at a resolution of 1 μm using a PC-controlled XYZ stage combined with a nano-positioning stage. A charged coupled device (CCD) connecting to the computer was installed above the objective lens to monitor the fabrication process in real time.

A layer-by-layer annular ablation from the top surface to internal substrate with 1 μm interval between the adjacent layers was adopted, so that the ablation always occurred at the interface between the water and the material. In this manner, the ablation debris can be more efficiently removed with the assistance of water. The laser power was chosen to be 0.35 mW for ablation in both the LN thin film and the LN substrate beneath the silica layer, whereas the laser power was raised to 1 mW for ablation in silica layer, because the ablation thresholds of LN crystal and silica glass are different.

In the FIB milling, a 30-kV ion beam with a beam current of 93 pA was used to polish the periphery of LN microdisk and waveguide.

### Measurement of Q-factor

The LN microdisk was tested using a narrow-band continuous-wave tunable diode laser (New Focus, Model 688-LN). The output laser from a single-mode fiber (SMF-28) was end-fire coupled into the waveguide using a 40× (NA 0.60) microscope objective, and the transmitted light was collected by a 100× (NA 0.80) microscope objective. By using an online fiber polarization controller, WGM modes in the microdisk with certain polarization were excited. A transient optical power detector (Lafayette, Model 4650) was used to measure the transmission spectra.

## Additional Information

**How to cite this article**: Fang, Z. *et al*. Monolithic integration of a lithium niobate microresonator with a free-standing waveguide using femtosecond laser assisted ion beam writing. *Sci. Rep.* **7**, 45610; doi: 10.1038/srep45610 (2017).

**Publisher's note:** Springer Nature remains neutral with regard to jurisdictional claims in published maps and institutional affiliations.
